# Gene identification and protein classification in microbial metagenomic sequence data via incremental clustering

**DOI:** 10.1186/1471-2105-9-182

**Published:** 2008-04-10

**Authors:** Shibu Yooseph, Weizhong Li, Granger Sutton

**Affiliations:** 1 J. Craig Venter Institute, 9704 Medical Center Drive, Rockville, MD 20850, USA; 2 California Institute for Telecommunications and Information Technology, University of California, San Diego, 9500 Gilman Drive, La Jolla, CA 92093, USA

## Abstract

**Background:**

The identification and study of proteins from metagenomic datasets can shed light on the roles and interactions of the source organisms in their communities. However, metagenomic datasets are characterized by the presence of organisms with varying GC composition, codon usage biases etc., and consequently gene identification is challenging. The vast amount of sequence data also requires faster protein family classification tools.

**Results:**

We present a computational improvement to a sequence clustering approach that we developed previously to identify and classify protein coding genes in large microbial metagenomic datasets. The clustering approach can be used to identify protein coding genes in prokaryotes, viruses, and intron-less eukaryotes. The computational improvement is based on an incremental clustering method that does not require the expensive all-against-all compute that was required by the original approach, while still preserving the remote homology detection capabilities. We present evaluations of the clustering approach in protein-coding gene identification and classification, and also present the results of updating the protein clusters from our previous work with recent genomic and metagenomic sequences. The clustering results are available via CAMERA, (http://camera.calit2.net).

**Conclusion:**

The clustering paradigm is shown to be a very useful tool in the analysis of microbial metagenomic data. The incremental clustering method is shown to be much faster than the original approach in identifying genes, grouping sequences into existing protein families, and also identifying novel families that have multiple members in a metagenomic dataset. These clusters provide a basis for further studies of protein families.

## Background

Biological sequence databases have continued to see an expansion in their size due to the large number of genome sequencing projects in the past few years. A large fraction of protein predictions submitted to databases are from microbial sequencing projects. Whole genome sequencing of bacteria, archaea, and viruses from various environments has provided clues to their adaptability and evolution. To-date, there are over 500 completed prokaryotic genomes, with an additional 800+ in various stages of completion [1]. However, the microbes that we have thus far been able to cultivate, study in the laboratory, and sequence, constitute only a small fraction (estimated to be <1%) of the microbes that exist in nature. This bottleneck is being addressed by the rapidly emerging area of metagenomics (or community genomics), where cultivation independent techniques are used to study the genomic sequences of organisms in a community [2]. In a typical metagenomic study, the DNA is extracted from a sample (collected from an environment of interest) and directly sequenced (for instance, using shotgun sequencing) [3,4]. Data from various metagenomic studies (for instance, [4-7]) have provided clues to the roles and interactions the constituent microbes play in their communities, and also have pointed to an incredible diversity of these organisms both at the genomic level and at the protein level. The recent Global Ocean Sampling (GOS) metagenomic study [8,9] to explore microbial diversity in the world's oceans, alone contributed more than 6 million protein predictions to the existing protein databases, thereby more than doubling the number of the then known proteins; in addition, these predictions were also shown to be a valuable resource for protein family studies by virtue of their diversity and novelty.

While metagenomic data are proving to be very useful in addressing evolutionary and ecological questions relating to microbial communities, they are also quite challenging to deal with [2,3,10,11]. Due to the current techniques used, the source organism of a metagenomic sequence is not known (unless there is an informative phylogenetic marker on the sequence). Furthermore, assemblies of metagenomic sequence data are typically fragmented. Several factors influence the assembly quality of a metagenomic sample, including the amount of sampling of the community, sequence coverage of individual organisms, and strain or sub-ribotype variation in the community [8,12]. Consequently, a large fraction of the protein sequences predicted in these data are fragmentary. Furthermore, gene-finding is also made challenging due to the presence of organisms that have varied GC compositions, codon biases etc. [10]. Several recent works have addressed these various challenges, including assembly [8], binning [13], gene identification [14,15], and protein classification [16].

The classification of proteins into families (usually based on their sequence similarity) serves the basis for further analyses of these families, including their structure and function [17,18]. Proteins are grouped together either on the basis of their domains [19,20] or on their full sequences [9,21].

In this paper, we present a computational improvement to a sequence clustering method that we introduced previously to analyze large microbial metagenomic datasets, and that was used in the GOS study [9]. This method could be used both to identify protein-coding genes in metagenomic data containing prokaryotic, viral and intron-less eukaryotic genomes, and to group related sequences into families (based on matches to the full sequence). However, this method requires the availability of similarities for all sequence pairs. This was computed in [9] using a BLASTP search [22], which becomes prohibitively expensive with the ever increasing amount of metagenomic sequence data that are being generated; in fact the all-against-all BLASTP searches of the 28.6 million sequences analyzed in the GOS study [9] required over 0.5 million CPU hours (on 3.06 GHz processors). In this paper, we present an incremental clustering approach that is much faster than the original approach. It does not use the all-against-all approach, but at the same time, preserves the homology detection capabilities of the earlier method. The method described here is currently used in CAMERA [23].

## Implementation

### Previous approach

We first provide a summary of our original clustering approach and the data sets used [9] so as to provide context to the method described in this paper. The original approach was intended to analyze the GOS microbial metagenomic data in the context of a comprehensive set of known proteins. Thus, data from other sources, namely, National Center for Biotechnology Information (NCBI)'s non-redundant amino acid database [24], NCBI's Prokaryotic Genome sequencing projects (PG) [24], Ensembl [25], and TIGR Gene Indices (TGI-EST) [26], were also included in the study. The input to the clustering consisted of full length and partial length amino acid sequences from these various data sources. For the nucleotide sequence sets GOS, PG, and TGI-EST, the corresponding amino acid sequence sets consisted of six frame translations, also known as Open Reading Frames (ORFs), identified on the nucleotide sequences. Only ORFs of length 60 amino acids or more were used in the study. To accommodate partial nucleotide sequences (in GOS and TGI-EST), the standard ORF definition was extended so that an ORF is bracketed by either a start codon or the start of the nucleotide sequence, and by either a stop codon or the end of the nucleotide sequence.

An all-against-all BLASTP compute was used to identify the pairwise sequence similarity used for the clustering. Given the size of the combined dataset (28.6 million amino acid sequences), for efficiency purposes, the clustering proceeded in a series of steps. First, a non-redundant set of sequences was identified from the combined data set. This step used pairwise matches with 90% identity (or 98% similarity) covering at least 95% of the shorter sequence length. In the second step, pairwise matches covering at least 80% of the longer sequence length were used to construct a graph of non-redundant sequences, and dense subgraphs were identified in this graph. Each dense subgraph is referred to as a *core cluster*, and corresponds to a sub-family of similar sequences. PSI-BLAST profiles [27] and FFAS profiles [28] were constructed for core clusters containing at least twenty sequences (using the longest core cluster sequence as query). The PSI-BLAST profiles were used to recruit singletons to core clusters, and the FFAS profiles were used to compare and merge related core clusters into final clusters.

Two filters were applied to the resulting final clusters to separate clusters of protein coding sequences from clusters of spurious sequences. The first filter (referred to here as the shadow ORF filter) identified shadow ORFs, that is, spurious ORFs that overlap with protein coding ORFs. The second filter (referred to here as the Ka/Ks filter) identified clusters of conserved but non-coding ORFs. These sequences show a lack of selection at the codon level and can be identified using their nonsynonymous to synonymous substitution ratios (Ka/Ks test) [14,29,30]. The two filters are also used in the incremental clustering method and will be described in more detail later.

### Input and strategy

The incremental clustering method has two inputs, a set *C *of (previously computed) protein clusters and a set *S *of amino acid sequences. *S *is a set of amino acid sequences from some protein resource, or in the case of metagenomic or genomic data analysis, *S *is the set of ORFs (length ≥60 amino acids is used here) identified from the nucleotide sequences (reads or contigs or scaffolds or chromosomes) as described earlier. Our method identifies and groups protein coding sequences into the existing protein clusters. In addition, it identifies novel protein families that have multiple members in *S*.

The incremental clustering method does not compare all sequences against each other. It does however incorporate varying homology detection capabilities. We use existing tools cd-hit (and its variant cd-hit-2d) [31-33], PSI-BLAST [27], and FFAS [28] to perform sequence-sequence, profile-sequence, and profile-profile comparisons respectively. Cd-hit is a fast sequence clustering algorithm that uses shared word counts as a filter to group highly similar sequences. Each cd-hit cluster is summarized by a cd-hit *representative *sequence, which, by construction, is also the longest sequence in the cluster. Cd-hit-2d, a variant of cd-hit, uses the same approach to identify sequences in a given set that are within a user-specified threshold to sequences in another set.

In the first stage of our incremental clustering method, cd-hit-2d is used to identify and recruit sequences in *S *that have high similarity (60% identity is used here) to sequences in *C*. In the second stage, the remaining sequences in *S *are clustered using cd-hit (at 60% identity). For both cd-hit-2d (Stage 1) and cd-hit (Stage 2), the 60% identity clustering is achieved in multiple steps rather than a single step – a high threshold 90% identity clustering step followed by a lower threshold 75% identity clustering step, and a final 60% identity clustering step. This is done for two reasons (efficiency and quality). Firstly, cd-hit and cd-hit-2d run much faster at a higher threshold (such as 90%) than at a lower threshold (60%). If *S *contains many sequences that have high identity to those in *C*, the initial faster high identity threshold clustering can recruit many of these sequences, thereby reducing the size of following slower runs. Secondly, in the current implementation of cd-hit, there are two modes for assigning a sequence to a cluster – assigning to the first cluster that meets the threshold (but which is not necessarily the best matching cluster for the sequence), and assigning to the best matching cluster. The current parallel version of cd-hit does not have the later option (which is the preferred option) implemented yet. In its absence, the multi-step approach provides a way to approximate the desired quality.

In the final stage, PSI-BLAST profiles for clusters in *C *and *S *are used to recruit sequences to these clusters. In addition, FFAS cluster profiles are used to merge groups of related clusters. As clusters get larger and more diverse with the addition of new data, cluster profiles provide better homology detection ability (compared to picking cluster representative sequences), and this is the rationale for using PSI-BLAST and FFAS profiles in the final stage of the incremental clustering. As in the original method, we also detect and remove clusters containing spurious sequences.

### Cluster organization and definitions

We define a sequence *s *to be *redundant *if it has a match with ≥90% identity to a longer sequence and this match covers ≥95% of the length of *s*; otherwise *s *is *non-redundant*. Following our previous work, every sequence is associated with a *core *cluster and a *final *cluster. One or more core clusters are grouped into a final cluster based on their FFAS profile matches. In the discussions below, a core cluster is labeled *big *if has ≥20 non-redundant sequences. Each big core cluster has a PSI-BLAST profile and an FFAS profile associated with it; currently, both profiles are computed using the longest sequence as query.

We assume that each cluster in *C *is made of one or more core clusters. We use *C-X *to denote the set of all cd-hit representatives computed from core clusters in *C *at the *X*% identity level. For cd-hit computes at the 90% identity described below, it is assumed that the matches are relative to the length of the *shorter *sequence, where as for lower thresholds (like 75% and 60% identities), it is assumed that the matches cover at least 80% length of the *longer *sequence.

### Incremental clustering method

**Figure 1:**
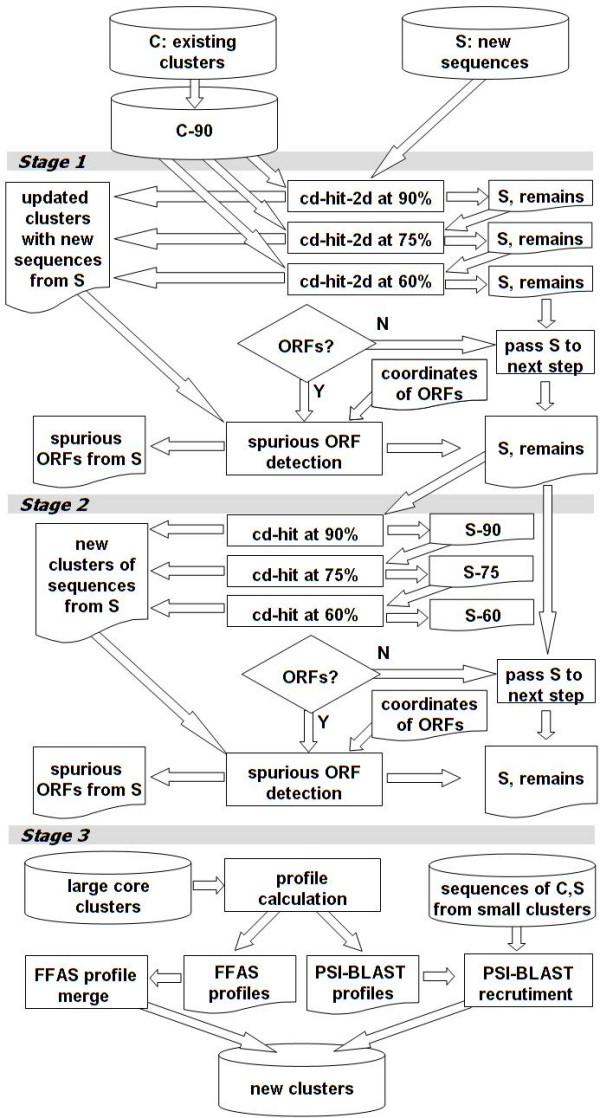
Flow chart of the incremental clustering method.

The incremental clustering method is shown in Fig. 1. It has three main stages.

### Stage 1: (Fast recruitment of *S *to *C*)

In this stage, sequences in *S *that are quite similar to sequences in *C-90 *are identified. For reasons discussed earlier, this is carried out in three steps. Sequences in *S *are first recruited to sequences in *C-90 *using cd-hit-2d at 90% identity. Subsequently, as yet unrecruited sequences in *S *are recruited to *C-90 *at 75% identity and then at 60% identity using cd-hit-2d. The sequences from *S *that are recruited in the previous two steps are then clustered again at 90% identity to identify the redundant sequences within them. After the recruitment step, PSI-BLAST and FFAS profiles are constructed for newly formed big core clusters in *C*. Also, profiles for those existing big core clusters that recruited sequences in *S *are refined. Finally, the shadow ORF filter is applied to *S *to identify and remove those unrecruited sequences in *S *that overlap (on the source read or contig or scaffold or chromosome) with the recruited sequences.

### Stage 2: (Clustering remaining sequences)

The purpose of this stage is to identify (core) clusters of similar sequences that remain in *S *after stage 1. Unrecruited sequences in *S *from the previous step are clustered successively at 90%, 75%, and 60% identities using cd-hit to produce core clusters. If *S *is a set of ORFs, then the shadow ORF filter and the Ka/Ks filter are used to identify clusters of spurious sequences. Finally, PSI-BLAST and FFAS profiles are computed for all newly formed big core clusters that are not labeled as spurious.

### Stage 3: (FFAS merging and PSI-BLAST recruitment)

Big core clusters are compared using their FFAS profiles. The comparisons are used to group these core clusters into final clusters. This is done by constructing a graph with nodes representing big core clusters. An edge exists between two nodes if the corresponding core clusters have a profile-profile match meeting a certain FFAS score threshold. We set the score thresholds as a function of the profile length, with profile lengths ≤500 set a threshold of -15, lengths >2000 set a threshold of -35, and lengths in between having thresholds between the two values. Each connected component in the constructed graph corresponds to a final cluster. Finally, PSI-BLAST profiles are used to recruit smaller clusters (including singletons) to big core clusters. A sequence is recruited to a big core cluster if the profile-sequence match has E-value ≤ 1e-8 (assuming database size of 1e9) and covers ≥75% of the sequence. A small cluster is recruited to a big core cluster if a majority of its sequences are recruited via PSI-BLAST to this big core cluster.

### Shadow ORF filter

In Stage 1, each unrecruited ORF in *S *that overlaps (on the source read or contig or scaffold or chromosome) with a recruited ORF is labeled as shadow and removed. Two ORFs on the same strand are considered overlapping if their intervals overlap by at least 60 bps. Two ORFs that are on the opposite strands are considered overlapping either if their intervals overlap by at least 50 bps and their 3' ends are within each others intervals, or if their intervals overlap by at least 120 bps and the 5' end of one is in the interval of the other. In Stage 2, a cluster is labeled as containing shadow ORFs if at least a third of its sequences overlap (with the same ORF overlapping criteria as before) with sequences in a bigger cluster.

### Ka/Ks filter

We use the Ka/Ks filter as described in [9]. For most proteins, Ka/Ks << 1, and for proteins that are under strong positive selection, Ka/Ks >> 1. A Ka/Ks value close to 1 is an indication that sequences are under no selective pressure and hence unlikely to code for proteins [29,34]. Weakly selected but legitimate coding sequences can have a Ka/Ks value close to 1. These are identified by using a model in which different partitions of the codons experience different levels of selective pressure. A cluster is rejected only if no partition is found to be under purifying selection at the amino acid level. The Ka/Ks filter is implemented as follows. Sequences in the cluster are first aligned with MUSCLE [35] and a strongly-aligning subset of sequences is selected for the Ka/Ks analysis. The codeml program from PAML [30,36] is run using model M0, to calculate an overall (i.e. branch- and position-independent) Ka/Ks value for the cluster. If Ka/Ks ≤0.5, the cluster is considered as passing the Ka/Ks filter (i.e. very likely coding). If not, the cluster is further examined by running codeml with model M3. This partitions the positions of the alignment into three classes that may be evolving differently (typically, a few positions may be under positive selection while the remainder of the sequence is conserved). A likelihood ratio test is applied to check if M3 explains the data significantly better than M0 [36]. If one of the resulting partitions has Ka/Ks ≤ 0.5 and comprises at least 10% of the sequence, then the cluster is considered as passing the Ka/Ks filter. If not, it is labeled as containing spurious ORFs.

### Clustering output

Final clusters that contain at least two non-redundant sequences and are not labeled as spurious (by one of the filters) are referred to as *good *clusters, and only sequences in these clusters are labeled as predicted proteins. Final clusters that contain only one non-redundant sequence but are not labeled as spurious, while not considered in the final statistics on predicted proteins, are kept around for future clustering.

## Results and Discussion

The sequences and clustering results from our previous study [9] that included NCBI-nr, Ensembl, TGI-EST, PG, and GOS, are used here as the starting point. Only those clusters (including those containing only one non-redundant sequence) that were not labeled as spurious are considered here. These clusters constitute the set *C*. We will refer to *C *as CAMERA clusters since this is currently available via the CAMERA effort [37]. This data set was updated using the incremental clustering method with all sequences that were submitted to various public protein databases since the data freeze used for [9]. The new sequences were collected via the Protein and Nucleotide Data Archive (PANDA) effort [38]. There were 2,955,580 amino acid sequences (with length ≥10 amino acids) in the release we used. In addition, the published data from the Hawaii Ocean Time series station ALOHA (HOT/ALOHA) metagenomic study [7] was also used. This study generated 65,674 sequencing reads from seven different ocean depths. We called 377,570 ORFs (length ≥60 amino acids) on these reads and this was also made available as input to our incremental clustering method. Thus the set *S *consisted of 3,333,150 amino acid sequences.

The hardware infrastructure consisted of a compute grid with 24 nodes, each with dual Xeon(TM) CPU 3.20 GHz, 4GB RAM; a total of 40 processors were available, with a varying number of them being used in the different stages of the incremental clustering.

Since the various steps of the incremental clustering process have different run time complexities, the actual total time (and also the individual component times) taken in an incremental update is dependent on the input data and their recruitment to the existing cluster data. An update of the CAMERA clusters with the nearly 3.3 million PANDA and HOT/ALOHA sequences took a total of 36.5 CPU days (or 877 CPU hours). For this dataset, the majority of the time was spent in the PSI-BLAST recruitment step (16 CPU days), followed by the cd-hit computations (13.9 CPU days) and the FFAS profile comparisons (6.5 CPU days). In contrast, an all pair sequence similarity computation of the 3.3 million sequences alone, via an all-against-all BLASTP search, on the same hardware is estimated to take 1,500 CPU days (or 36,000 CPU hours).

We used simulated data to evaluate the gene identification capability of the clustering approach on unassembled read sequences (with lengths corresponding to those of sequences generated by the Sanger method [39]). An evaluation of a homology-based gene identification method (such as ours) in a metagenomic setting has the additional complexity of accurately modeling the population structure of the microbial community. This is relevant in the context of identifying novel protein families that may be specific to a particular taxonomic group represented in the metagenomic sample. Microbial population structures vary from environment to environment, and even over time in a given environment. For the current evaluation, we chose to avoid the population modeling issue and directly address gene identification on fragmentary sequences. Sequence reads were generated from seventeen recent genome projects (Table 1). Data from these genome projects were not available at the time of construction of the CAMERA clusters [9]. The list in Table 1, however, includes both closely related and distantly related organisms to those that contributed to the CAMERA clusters. Sequence reads of length 800 bp were randomly generated from each genomic sequence; a 2× coverage (rather than 1× coverage) was assumed in the generation so as to sample a larger number of genes in the genome. ORFs (i.e. six frame translations) were generated from these reads using translation table 11; only ORFs of length ≥60 aa were considered. This resulted in a total of 513,267 ORFs from the seventeen genome projects. Out of these, 160,731 ORFs overlapped (in the same reading frame) with a gene; we refer to this set as the *reference set*. The 513,267 ORFs constituted the input *S *to the incremental clustering method (with *C *being the CAMERA clusters). Our method labeled 127,256 ORFs as protein coding, of which 119,864 were in the reference set. A total of 2,829 ORFs (1.7%) from the reference set were incorrectly labeled as spurious by the filters.

**Table 1:** Sensitivity and Specificity of gene identification using the incremental clustering method.

**Genome**	**Kingdom**	**%GC**	**%Sn**	**%Sp**
Acaryochloris marina MBIC11017	B	47.2	70	96.4
Acidobacteria bacterium Ellin345	B	58.3	68.3	95.9
Acidiphilium cryptum JF-5	B	67.9	80.9	84
Acinetobacter baumannii ATCC 17978	B	38.9	80.6	95.5
Alcanivorax borkumensis SK2	B	54.7	84.7	97.7
Bacteroides vulgatus ATCC 8482	B	42.2	73.3	97
Burkholderia thailandensis E264	B	67.2	81.1	87.5
Caldivirga maquilingensis IC-167	A	43	67.3	97.8
Candidatus Methanoregula boonei 6A8	A	54.5	67.1	95.6
Candidatus Pelagibacter ubique HTCC1062	B	29.6	98.1	98
Fervidobacterium nodosum Rt17-B1	B	34.9	76.3	97.1
Francisella tularensis subsp. Holarctica	B	32.1	83.2	87.7
Hyperthermus butylicus DSM 5456	A	53.7	61.3	94
Lactobacillus salivarius UCC118	B	32.9	78.4	93
Methanococcus aeolicus Nankai-3	A	30	73.8	97.8
Staphylothermus marinus F1	A	35.7	63.8	96.7
Thermofilum pendens Hrk 5	A	57.6	63.9	97.4
*Average*			74.8	94.7

A-Archaea, B-Bacteria, Sn-Sensitivity, Sp-Specificity.

Our method has an average specificity of 94.7% and an average sensitivity of 74.8% on these genomes (Table 1). The evaluation highlights several aspects of the method. First, our method, by design, has high specificity. This is a result of the conservative constraint that we use to label an ORF as a predicted protein (namely, that it must belong to a cluster that contains at least two non-redundant sequences and that is not labeled as spurious by the filters). Second, the sensitivity of our homology-based method in detecting a new organism's genes is dependent on the representation in the existing protein clusters *C *(and in the set *S*) of this organism's taxonomic neighbours. For instance, our method has very high sensitivity (98.1%) on *Candidatus Pelagibacter ubique HTCC106 *[40], an alphaproteobacteria that is present in ocean surface waters and is well represented in the GOS data (even though an assembly of this organism's genome could not be inferred from the GOS data due to various reasons including the tremendous sub-ribotype variation) [8]. On the other hand, our method has a much lower sensitivity (68.3%) on a soil bacterium *Acidobacteria bacterium Ellin345 *[41], which belongs to the class acidobacteria; this class is not well represented in *C*. The sensitivity numbers of our method on these genomes also have to be placed in the context of the number of ORFans [42] seen in newly sequenced genomes. ORFans are protein predictions that have no homology to known proteins. They have been seen to account for 25–30% of protein predictions in newly sequenced prokaryotic genomes [43]. Our method will not label ORFan sequences as proteins since they will fall into singleton clusters. The number of ORFans will no doubt decrease as more related genomes from similar environments are sequenced. Our approach retains these sequences for future clustering. Finally, Table 1 also shows lower sensitivity numbers for archaea (66.2%) compared to bacteria (79.5%). This is a consequence of a much sparser sampling (i.e. genomes sequenced) of archaea compared to bacteria, and therefore a relatively smaller representation of archaea in the existing protein clusters *C*.

We also compared the performance of our clustering approach to that of a non-homology based genefinder (MetaGene [15]) using two metagenomic datasets, namely, the GOS data and the HOT/ALOHA data. From the input 17,422,766 GOS ORFs [9], our clustering approach identifies 6,121,630 ORFs as protein coding whereas MetaGene (run on the GOS assemblies that served as the source of the ORF set) produces predictions that can be mapped (i.e. overlaps with and is in the same reading frame) to 6,424,656 GOS ORFs. The two sets have 5,647,789 predictions in common (that is, 92% of cluster predictions and 88% of MetaGene predictions). We analyzed how many of the predictions unique to each method had matches to models in the Pfam database [19], since this database is widely used for functional annotation. Of the 776,867 MetaGene-only predictions, 32,004 (4%) have matches (with trusted cutoffs and E-value ≤ 1e-3) to Pfam models, whereas of the 473,841 clustering-only predictions, 100,914 (21%) have matches to Pfam models. On the HOT/ALOHA data, 57,333 of the input 377,570 ORFs are labeled as protein coding by our clustering. MetaGene (run on the read set that served as the source of the ORF set) produces predictions that can be mapped to 71,599 ORFs. There are 46,691 predictions in common to the two sets (that is, 81% of cluster predictions and 65% of MetaGene predictions). Of the 24,908 MetaGene-only predictions, 1387 (6%) of the sequences have matches to Pfam models, whereas of the 10,642 clustering-only predictions, 3311 (31%) have matches to Pfam models.

Comparisons to MetaGene on the two datasets reveal common patterns. While both approaches agree on a large fraction of their predictions, MetaGene makes more predictions than our clustering approach, and this can be explained as follows. While it is possible for MetaGene to identify novel families even if only a single member is present, our conservative approach requires that multiple (and non-redundant) members of a novel family are present. Thus, we do not make protein predictions for these sequences that fall into singleton clusters. As previously stated, we do, however, retain these ORF sequences for future clusterings and resolution. A comparison of predictions unique to each method using Pfam models reveals that a larger fraction of predictions unique to our approach (compared to Metagene's) have Pfam matches. This is a consequence of using a homology-based approach.

**Figure 2:**
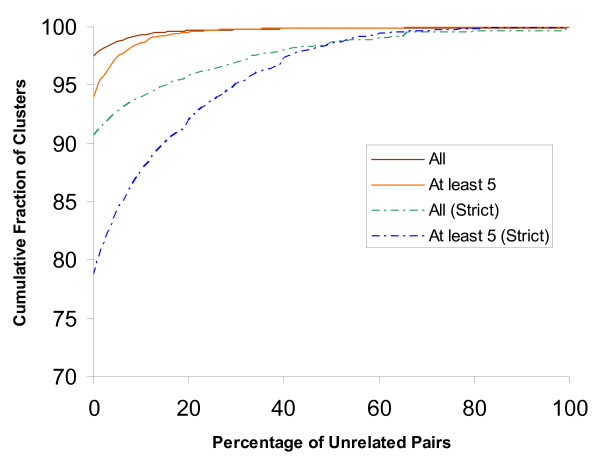
**Percentage of Unrelated Pairs in Clusters**. For all clusters, only Pfam match-containing sequences were considered. For the top curve (labeled All), all clusters with at least two Pfam match-containing sequences were considered where as for the second curve (labeled At least 5), only those clusters with at least five Pfam match-containing sequences were considered. For the later curve, it is seen that 94% of the reported clusters have no unrelated pairs. The bottom two curves show the trends for the "strict" version of unrelatedness.

**Figure 3:**
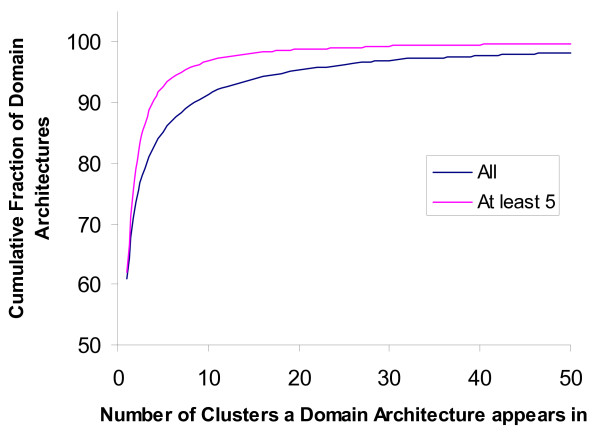
**Number of clusters that domain architectures appear in**. For the bottom curve (labeled All), all domain architectures were considered whereas for the top curve a domain architecture is considered as appearing in a cluster only if it has at least five instances in that cluster. In both cases, nearly 61% of domain architectures appear in a single cluster, and over 80% of domain architectures appear in at most 3 clusters.

We evaluated our clustering methodology in its ability to classify sequences into protein families. The lack of availability of an exhaustive data set that can be used as a gold standard to evaluate large scale computational protein classification (based on full length sequences), presents its own challenges. For our evaluations we used the domain architecture based approach [9], which is an attempt to get at the full length matches using the Pfam domain matches. We used the Pfam results from [9] together with Pfam results on the HOT/ALOHA set, and restricted our analysis to those clusters that contain sequences with Pfam matches. Briefly, the domain architecture for a sequence is defined to be the set of all Pfams that have (trusted) matches to it. Two sequences are defined to be *unrelated *if their domain architectures each have at least one Pfam that is not present in the other's domain architecture. We also consider here a *strict *version, where two sequences are considered unrelated if either their domain architectures have no Pfams in common, or when they do, then they each have at least one Pfam that is not present in the other's domain architecture. Using these definitions, we plotted the cumulative fraction of (final) clusters against the percentage of unrelated pairs they contain (Fig. 2). These curves show that our clustering is quite consistent, that is, the clusters have a low fraction of unrelated sequence pairs; 98% of all clusters have no unrelated pairs (this number drops to 94% if only those clusters with at least five Pfam-match containing sequences are considered). For the strict version, 91% of all clusters have no unrelated pairs (this number drops to 79% if those only clusters with at least five Pfam-match containing sequences are considered). We also evaluated how often domain architectures are split across (final) clusters (Fig. 3) and found that over 80% of the domain architectures appear in three or fewer clusters.

**Figure 4:**
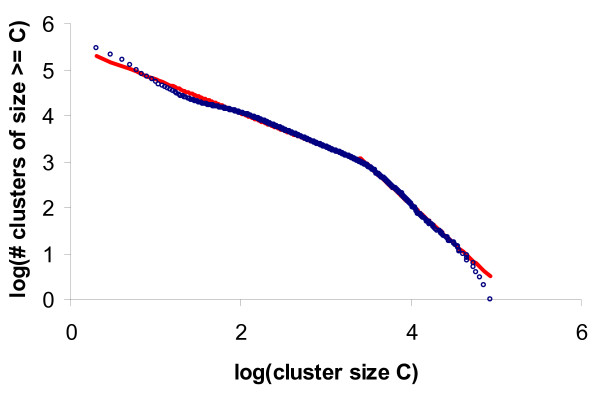
**Log-Log Plot of Cluster Size Distribution**. The x-axis is the logarithm of the cluster size C and the y-axis is the logarithm of the number of clusters of size ≥C. Logarithms are in base 10. The blue curve is the observed data, which is consistent with a power law. There is an inflection point around C = 2500 (a value of 3.4 on the x-axis). The two red lines are the least square fit to C ≤ 2500 and C > 2500, respectively. The former line is y = -0.733*x + 5.517, with R^2 ^= 0.995, and the later line is y = -1.686*x + 8.813, with R^2 ^= 0.992.

From the input sequence set, 2,464,046 PANDA sequences (83%) and, as mentioned previously, 57,333 HOT/ALOHA sequences (15%) are labeled as predicted proteins. The incremental clustering update resulted in 284,297 good clusters; see Table 2 for cluster size distribution. These final clusters contained a total of 12,725,982 sequences, with nearly 88% of the sequences in 17,164 clusters that contain at least twenty non-redundant sequences. Fig. 4 shows the Log-Log plot of final cluster size distribution to be consistent with a power law. As noted in previous studies [9,44], the observed curve has an inflection point showing differing power laws governing the size distribution of very large clusters compared to the rest.

**Table 2:** Cluster size distribution and the distribution of sequences in these clusters

**Cluster size**	**#clusters**	**#sequences**	**#non-redundant sequences**
2–4	208,096	794,592	521,898
5–9	43,453	428,469	273,694
10–19	15,584	346,415	206,188
20–49	4,053	234,338	143,438
50–99	4,641	547,862	331,773
100–199	3,546	870,406	491,229
200–499	2,600	1,381,135	806,560
500–999	961	1,133,749	669,420
1,000–1,999	698	1,768,532	1,002,815
≥2,000	665	5,220,484	2,909,845
Total	284,297	12,725,982	7,356,860

The size of a cluster is defined as the number of non-redundant sequences in it.

The PANDA sequences that are labeled as predicted proteins (i.e. belonging to good clusters) have a great deal of redundancy. Of these 2,464,046 sequences, 1,528,382 (62%) are marked as redundant, with 1,353,885 (55%) being marked as redundant by existing cd-hit representatives in the CAMERA clusters and the remaining being marked as redundant by other PANDA sequences. An examination of those PANDA sequences that are not labeled as predicted proteins by our clustering reveals that 379,021 (77%) have a hypothetical or unknown in their sequence headers; these sequences may be organism specific proteins, which our current method will not identify, or they may be spurious protein predictions submitted to the public databases. The good clusters containing the largest number of PANDA sequences include reverse transcriptases, cytochromes, ABC transporters, and dehydrogenases (see Table 3).

**Table 3:** Clusters recruiting largest number of PANDA sequences

**Cluster ID**	**#sequences**	**#non-redundant sequences**	**Description**
CAM_CL_2057	20,508	24	Reverse transcriptase (HIV)
CAM_CL_1132	18,882	1,406	Cytochrome c oxidase subunit I
CAM_CL_2568	15,405	6,091	ABC transporter
CAM_CL_4367	15,228	771	Cytochrome b
CAM_CL_49	14,751	7,389	Short-chain dehydrogenase
CAM_CL_3510	13,255	5,173	Immunoglobulin
CAM_CL_2630	13,140	3,297	Envelope glycoprotein
CAM_CL_160	13,054	3,897	Kinases
CAM_CL_4556	12,403	6,345	Response regulator
CAM_CL_481	12,078	5,477	Transcription regulator

Column 3 hints at the extent of redundancy in the PANDA set.

Compared to the PANDA set, the HOT/ALOHA predicted protein sequences show a lesser amount of redundancy. 7,958 sequences (14%) are marked as redundant, with 6901 (12%) being marked as redundant by existing cd-hit representatives from the CAMERA clusters. A breakdown of the predicted proteins by their sample depths reveals differential abundances of many protein families. Proteins involved in bioluminescence and families of transposases and integrases are more abundant with depth whereas proteorhodopsins and photolyases are more abundant in the surface and near surface water samples. These differences are a reflection of the environmental factors that shape microbial communities and have been noted previously [7]. Table 4 lists the clusters containing the largest number of HOT/ALOHA sequences.

**Table 4:** Clusters recruiting largest number of HOT/ALOHA sequences

**Cluster ID**	**# sequences**	**Process, Protein Family**
CAM_CL_49	562	Metabolism, short chain dehydrogenase
CAM_CL_399	368	Metabolism, Sulfatase
CAM_CL_26	338	electron transport, Acyl-CoA dehydrogenase
CAM_CL_1239	314	metabolism, AMP-binding enzyme
CAM_CL_2568	312	transport, ABC transporter
CAM_CL_1581	274	bioluminescence, methanogenesis, Luciferase-like monooxygenase
CAM_CL_4294	240	nucleotide-sugar metabolism, NAD dependent epimerase/dehydratase family
CAM_CL_1593	235	metabolism, CoA-transferase family III
CAM_CL_357	227	Tetratricopeptide repeat
CAM_CL_333	225	lignin biosynthesis, Zinc-binding dehydrogenase

In [9], we reported on novel protein clusters (the so called Group II clusters) from the GOS data that could not be linked to any of the then known families (via any of the remote homology methods used). We explored these clusters in the context of the current incremental data set. The PANDA set included protein predictions from recently sequenced microbes, including several marine prokaryotes that were sequenced by the Gordon and Betty Moore Foundation sponsored projects [45]. 552 of the originally labeled Group II clusters had at least one PANDA or HOT/ALOHA sequence, with 35 containing ≥10 of them. Table 5 lists the genome projects present in the PANDA set that have the largest number of sequences in these clusters. This table shows that most of them are Moore sponsored projects. It may not be surprising that most of the recruitment to the GOS-only clusters is from new microbial sequences from the marine environment. Nevertheless, these recently sequenced genomes can provide useful anchors to carry out further analyses of these protein families that could eventually provide clues to their functions and evolution.

**Table 5:** Recent genome projects with protein predictions that fall in Group II clusters.

**Genome project**	**#sequences recruited**
Psychroflexus torquis ATCC 700755^a^	110
Cellulophaga sp. MED134^a^	38
Flavobacteriales bacterium HTCC2170 ^a^	36
Robiginitalea biformata HTCC2501^a^	32
Croceibacter atlanticus HTCC2559 ^a^	31
Gramella forsetii KT0803	31
Leeuwenhoekiella blandensis MED217 ^a^	31
Flavobacterium johnsoniae UW101	29
Polaribacter irgensii 23-P ^a^	26
Tenacibaculum sp. MED152 ^a^	25
Flavobacteria bacterium BBFL7 ^a^	22
Bacteriophage Syn9	18
Microscilla marina ATCC 23134 ^a^	18
Marine gamma proteobacterium HTCC2080^a^	17
Candidatus Pelagibacter ubique HTCC1002^a^	12
Magnetospirillum magneticum AMB-1	12
Marine gamma proteobacterium HTCC2143 ^a^	10
Prochlorococcus marinus str. MIT 9312	10
Alpha proteobacterium HTCC2255 ^a^	9
Marine gamma proteobacterium HTCC2207^a^	9

^a ^Marine Microbial Genome projects funded by the Gordon and Betty Moore Foundation

The incremental clustering data is available for download from the Publications and Data section at CAMERA [37].

## Conclusion

We presented an incremental clustering method that is a computational improvement to an earlier method to identify and classify proteins in large microbial metagenomic datasets. The resulting clusters can serve as the basis for further analyses including functional annotation and evolutionary studies of different protein families.

Our method can be applied to metagenomic data sets that contain prokaryotes, viruses, and intron-less eukaryotic genomes. It has been applied to data generated by the Sanger sequencing technology [39], where current read lengths are ~800 bp. Innovations in sequencing technologies have resulted in several recent approaches that are cheaper and produce more sequence data (per run) compared to Sanger sequencing [46]. These next generation sequencing (NGS) methods, including the pyrosequencing based technology of 454 Inc [47] that currently produces reads of length ~250 bp, have higher sequence read error rates (as much as 3%). The high error rates can result in insertions or deletions in the nucleotide sequence that produce shifts in the reading frame, thereby adding to the complexity of the gene identification process. The ORF generation based method described in this paper cannot be directly applied to these read data. They could, however, be applied to assembled contigs that have high coverage, and subsequently much smaller error rates. We are also currently developing an incremental clustering approach that does not require explicit generation of six frame translations to identify genes from these data. The extent and accuracy of gene calling and protein classification on data generated by other NGS methods that produce shorter reads (30–100 bp) also needs to be explored.

An evaluation of our clustering method in identifying genes showed that the method has high specificity. The sensitivity of the method can be increased by developing ORF confidence measures (based on GC composition and codon usage) [48] for sequences in singleton clusters. We also compared the performance of our clustering method to MetaGene in identifying genes in metagenomic datasets. While MetaGene makes more gene calls and is fast, our method takes a more conservative approach to identifying protein coding sequences (by requiring multiple evidence) and at the same time, also groups related sequences into families. Based on an evaluation using the Pfam database, we also found that, compared to MetaGene, our homology-based method tends to pick up a larger fraction of sequences with matches to known protein families. Thus, a metagenomic annotation system will benefit from making use of both types of approaches.

Future modifications to improve the specificity and sensitivity of the clustering method will include alternate ways of constructing PSI-BLAST and FFAS profiles (for instance, using a centroid sequence as a query, rather than the currently used longest sequence, to construct profiles, and possibly representing core clusters that begin to show a lot of divergence, with multiple profiles), and also approaches to detect and handle over- and under- clustering. Even though we have presented our approach as an analysis tool for microbial metagenomic data, it is also applicable to analyzing finished or nearly finished prokaryotic genome projects. The clustering information presented here will be periodically updated with data from newer prokaryotic genome and metagenome projects. Cluster annotation and linking to other already existing valuable protein resources, is currently being done, and will also be made available.

## Availability and requirements

Cd-hit, PSI-BLAST, FFAS, MUSCLE, and PAML are important components of our incremental clustering approach. They are all published methods, and their availability and requirements are described at their respective homepages: Cd-hit , PSI-BLAST , FFAS , MUSCLE  and PAML . The shadow ORF filter code and the Ka/Ks filter code are available for download from the Publications and Data section at CAMERA . Operating system: Linux; Programming languages: Perl and C; License: GNU GPL.

## Authors' contributions

SY and WL contributed to the design, implementation, validation of methods, analysis of results, and writing of manuscript. GS contributed to design and validation of methods.
